# Development and psychometric validation of the NICU parental agency scale: a multicenter cross-sectional study

**DOI:** 10.3389/fped.2026.1842011

**Published:** 2026-07-27

**Authors:** Li Chen, Lili Li, Wenying Zhu, Jie Zhang, Zhen Wang, Yingwen Zhu, Yuan Gu, Shenzhen Zhang, Xin Ding, Yun Yu

**Affiliations:** 1 Department of Neonatology, Children's Hospital of Soochow University, Suzhou, China; 2 Neonatology Department, Suzhou Municipal Hospital, Suzhou, China; 3 Neonatology Department, The First People's Hospital of Kunshan, Kunshan, China; 4 Neonatology Department, The Ninth Affiliated Hospital of Soochow University, Suzhou, China; 5 Neonatology Department, Suzhou Hospital of Integrated Traditional Chinese and Western Medicine, Suzhou, China; 6 Neonatology Department, Changshu NO.1 People's Hospital, Changshu, China; 7 Department of Pediatrics, The First People's Hospital of Taicang City, Taichang, China

**Keywords:** caregiver engagement, family-centered care, NICU, parental agency, psychometric validation, scale development

## Abstract

**Background:**

Admission to the neonatal intensive care unit (NICU) places families in complex medical environments where parental agency, the perceived capacity to understand treatment, communicate effectively, participate in decisions, and maintain emotional well-being, is crucial for family-centered care. This study aimed to develop and psychometrically validate the NICU Parental Agency Scale (NICU-PAS), a brief instrument measuring this multidimensional construct.

**Methods:**

The 15-item NICU-PAS was developed through literature review, expert panel evaluation, and parent focus groups. A multicenter cross-sectional study was conducted across eight NICUs in Suzhou, China, recruiting 350 primary caregivers. Psychometric validation included exploratory and confirmatory factor analysis, internal consistency reliability, and assessment of convergent validity (with parental stress) and discriminant validity (with general health and coping). Subgroup analyses compared first-time and experienced parents.

**Results:**

Confirmatory factor analysis supported the four-domain structure of Treatment Understanding, Communication, Decision Making Participation, and Emotional Well-being (χ^2^/df = 1.69, CFI = 0.972, RMSEA = 0.045). Internal consistency was good (Cronbach's α = 0.783–0.860 for domains; 0.839 for total scale). Convergent validity was demonstrated by a significant negative correlation with parental stress (*r* = −0.189, *p* < 0.001), while discriminant validity was supported by negligible associations with general health and coping (*r* < 0.05). Experienced parents reported higher agency than first-time parents in Communication (*p* = 0.013), Emotional Well-being (*p* = 0.005), and total scores (*p* = 0.002).

**Conclusions:**

The NICU-PAS demonstrates robust psychometric properties and provides a practical, domain-specific measure of parental agency suitable for research and clinical quality improvement in NICU settings.

## 1 Introduction

Admission of an infant to the neonatal intensive care unit (NICU) places families in a situation marked by medical complexity, uncertainty, and rapid decision-making. Caregivers are expected to absorb unfamiliar clinical information, communicate with multiple members of the care team, and make value-sensitive choices under time pressure, often while experiencing marked emotional distress. In this context, family engagement is not only a psychosocial concern but also a practical component of care delivery, shaping how effectively information is exchanged, how decisions are understood, and how caregivers participate in ongoing treatment planning (1–4).

Within NICU care, the concept of parental agency captures a caregiver's perceived capacity to act effectively in relation to their infant's hospitalization. Parental agency in the NICU can be understood as the extent to which caregivers feel able to comprehend treatment information, communicate with clinicians, participate in decisions, and maintain emotional functioning sufficient to stay engaged (5–8). This construct is closely aligned with the goals of family-centered care because it reflects not only what clinicians provide but also what caregivers feel capable of doing in response (3, 9, 10). Importantly, agency is conceptually distinct from related outcomes such as stress, anxiety, depression, satisfaction with care, or general coping style (11–14). A caregiver may report high stress while still feeling able to ask questions, understand the care plan, and participate in decisions. Conversely, low perceived agency may persist even when global satisfaction is acceptable, particularly when information is difficult to interpret or communication is constrained (1, 2, 14).

A range of instruments has been used to assess caregiver experiences in pediatric and neonatal settings, including measures of parental stress, psychological symptoms, satisfaction, and broad empowerment (15–17). However, many commonly used NICU-focused tools emphasize stressors and emotional burden rather than the caregiver's perceived ability to engage in day-to-day care processes (15, 17). Instruments developed for empowerment or activation in other populations often do not directly map onto the operational tasks that families face during NICU hospitalization, such as understanding complex treatment plans, navigating clinical communication, and participating in decisions (18, 19). As a result, existing measurement approaches may not adequately capture the actionable aspects of caregiver experience that could inform quality improvement and targeted interventions within NICU services.

The literature on family-centered neonatal care, parent participation, shared decision-making, and psychosocial adjustment indicates that several care-process dimensions are central to parental agency in the NICU, particularly treatment understanding, communication with clinicians, participation in decision-making, and emotional well-being (18–23). Treatment Understanding reflects whether caregivers feel capable of following their infant's condition and care plan (19, 22). Communication reflects whether caregivers feel able to ask questions and obtain clear responses (20, 22). Decision Making Participation reflects whether caregivers feel included in choices and able to express preferences (18, 20). Emotional Well-being reflects whether caregivers feel sufficiently supported and emotionally stable to remain engaged during hospitalization (21, 23). A domain-based framework grounded in these care processes offers practical value because it distinguishes between specific areas where caregivers may experience difficulty and where NICU teams may intervene through education, communication strategies, shared decision-making support, or psychosocial care (15, 18).

A brief, NICU-specific, psychometrically sound measure of parental agency can provide a common language for research and for the evaluation of family-centered care practices. Such a measure should be short enough for routine use, reflect domains that correspond to common NICU interactions, and demonstrate reliability and validity. To meet this need, the NICU Parental Agency Scale (NICU-PAS) was developed through a structured process that integrated evidence from the literature, expert review by neonatal care professionals, and qualitative feedback from parents with NICU experience. The resulting instrument was designed as a concise 15-item scale representing four theoretically derived domains: Treatment Understanding, Communication, Decision Making Participation, and Emotional Well-being, with items rated on a 5-point Likert scale.

The present study aimed to develop and psychometrically validate the NICU-PAS in a multi-center sample of caregivers recruited from eight NICUs in Suzhou, China. The primary objectives were to evaluate internal consistency reliability for each domain and the total scale, examine the latent structure using exploratory factor analysis (EFA), and confirm the identified structure using confirmatory factor analysis (CFA). Construct validity was assessed by examining convergent validity through associations with parental stress and by testing discriminant validity using constructs expected to show minimal association with parental agency. In addition, subgroup comparisons were conducted to explore whether the NICU-PAS could detect differences between first-time and experienced parents, providing initial evidence of sensitivity to meaningful variation in caregiver experience.

Based on the conceptual framework used in scale development, a four-domain structure was expected, reflecting treatment understanding, communication, decision-making participation, and emotional well-being (15, 18). It was anticipated that domain and total scores would demonstrate acceptable to good internal consistency (24, 25). For convergent validity, higher perceived parental agency was expected to be associated with lower parental stress (21, 26). For discriminant validity, weak or non-significant correlations were expected between NICU-PAS scores and constructs with limited theoretical overlap, such as general health ratings and a brief coping checklist (25, 26). Finally, parenting experience was expected to be associated with differences in perceived agency in at least some domains, given the role of prior experience in navigating healthcare interactions and managing uncertainty during an infant's hospitalization (23, 27).

By developing and validating a brief, domain-based measure of parental agency tailored to NICU care processes, this study seeks to support more precise assessment of caregiver engagement and capacity during NICU hospitalization. A psychometrically supported measure of NICU parental agency can facilitate research on caregiver outcomes, enable evaluation of interventions designed to improve communication and family support, and guide targeted improvements in NICU practice by identifying specific domains in which caregivers report lower agency (22).

## 2 Materials and methods

### 2.1 Study design and participants

This study employed a multi-center, cross-sectional design conducted at eight NICUs in Suzhou, China. The target population comprised parents, guardians, and primary caregivers of infants currently admitted to the NICU. Participants were recruited using a convenience sampling method over a three-month period.

Inclusion criteria for participation were as follows: (1) individuals aged 18 years or older; (2) identified as the primary caregiver of the infant; (3) infant had been admitted to the NICU for a minimum of 72 h to ensure sufficient exposure to the NICU environment; and (4) ability to read and understand Chinese. Exclusion criteria included: (1) caregivers with documented cognitive impairments or psychiatric conditions that would impede comprehension of the survey; (2) caregivers whose infants were in critical condition with imminent risk of mortality; and (3) non-primary caregivers or temporary guardians.

The target sample size was established at 250–300 participants, with an anticipated non-response rate of approximately 10%. To ensure adequate statistical power for the planned psychometric analyses, a total of 350 participants were recruited. Research assistants approached eligible caregivers during designated visiting hours, explained the study purpose, and obtained informed consent.

### 2.2 Instrument development

The development of the NICU Parental Agency Scale (NICU-PAS) followed a systematic, multi-stage approach grounded in established scale development methodology. The initial stage involved a comprehensive literature review of existing instruments measuring parental agency, patient empowerment, and family-centered care in pediatric and neonatal settings. Key instruments reviewed included the Family Empowerment Scale, the Patient Activation Measure, the Parental Stressor Scale: Neonatal Intensive Care Unit, and published NICU-focused measures of family-centered care, parent participation, stress, and coping (15–28). Items in the preliminary pool were adapted conceptually from these instruments and from the NICU family-centered care literature rather than copied verbatim. Items addressing caregiver confidence, communication, and participation were informed primarily by empowerment, activation, and family participation measures, whereas items addressing emotional strain and coping during hospitalization were informed by NICU parental stress and coping instruments.

Subsequently, an expert panel comprising five professionals with specialized expertise in neonatal care was convened. The panel included two neonatologists, two NICU nurses with family-centered care training, and one clinical psychologist specializing in perinatal mental health. Panel members evaluated the initial 28-item pool for content validity, relevance to NICU contexts, clarity of wording, and comprehensiveness of domain coverage. Items were rated on a 4-point scale (1 = not relevant, 4 = highly relevant), and content validity indices were calculated. Items with content validity indices below 0.78 were either revised or eliminated.

Following expert review, focus groups were conducted with 12 parents (six mothers and six fathers) who had recent NICU experience. These semi-structured discussions explored caregivers' experiences with treatment understanding, communication with healthcare providers, participation in decision-making, and emotional well-being during their infant's NICU stay. Parent feedback was used to assess item clarity, acceptability, redundancy, and relevance to real NICU experiences. After this final refinement step, seven additional items were removed because they were judged to overlap substantially with retained items, were difficult for parents to interpret consistently, or did not add distinct content beyond the four intended domains. The item refinement process therefore incorporated evidence from the literature review, expert panel review, and parent focus groups, resulting in a final 15-item scale comprising four theoretically derived domains: Treatment Understanding (4 items), Communication (4 items), Decision Making Participation (4 items), and Emotional Well-being (3 items). The final questionnaire items and scoring instructions are provided in Appendix A.

### 2.3 Psychometric validation

#### 2.3.1 Reliability

Internal consistency reliability was assessed using Cronbach's alpha coefficient for each domain and the total scale. An alpha coefficient greater than 0.70 was considered acceptable for research purposes, while values exceeding 0.80 were considered good. Additionally, corrected item-total correlations were examined to identify any items that did not correlate adequately with the total scale score. Items with item-total correlations below 0.30 were flagged for potential removal or revision.

#### 2.3.2 Factor structure

The factor structure of the NICU-PAS was evaluated using both exploratory and confirmatory factor analysis. Exploratory factor analysis (EFA) was conducted to examine the underlying structure of the NICU-PAS. Principal axis factoring was used for factor extraction, and factors were rotated using varimax rotation to aid interpretability and item grouping. Sampling adequacy was evaluated using the Kaiser-Meyer-Olkin (KMO) statistic and Bartlett's test of sphericity. The number of factors retained was determined using eigenvalues greater than 1.0, inspection of the scree plot, and theoretical interpretability. Items with factor loadings ≥0.40 were considered to load meaningfully on a factor. Correlations among resulting domains were examined separately.

Confirmatory factor analysis (CFA) was conducted using AMOS software (version 24.0) to validate the factor structure identified in the EFA. Maximum likelihood estimation was employed given the approximately normal distribution of item responses. Model fit was evaluated using multiple indices: the chi-square to degrees of freedom ratio (χ^2^/df), comparative fit index (CFI), Tucker–Lewis index (TLI), root mean square error of approximation (RMSEA) with 90% confidence intervals, and standardized root mean square residual (SRMR). Acceptable model fit was defined as χ^2^/df < 3.0, CFI and TLI > 0.90, RMSEA < 0.08, and SRMR < 0.08.

#### 2.3.3 Convergent and discriminant validity

Convergent validity was assessed by examining correlations between the NICU-PAS and theoretically related constructs. The NICU Parental Stress Scale (NICU-PS), a validated 28-item instrument measuring stress experienced by parents of NICU infants, was administered concurrently. Based on theoretical considerations, a negative correlation was expected, such that higher levels of parental agency would be associated with lower levels of parental stress.

Discriminant validity was evaluated by examining correlations with measures that should demonstrate minimal theoretical overlap with parental agency. These included a single-item general health rating and a brief coping strategies checklist. Low or non-significant correlations with these measures would support the discriminant validity of the NICU-PAS.

### 2.4 Sample size estimation

Sample size requirements were determined based on established guidelines for psychometric validation studies. For exploratory factor analysis, a minimum of 5–10 participants per item is recommended. With 15 items in the NICU-PAS, this yielded a minimum required sample size of 75–150 participants. For confirmatory factor analysis and reliability analyses, larger samples of 250–300 participants were targeted to ensure stable parameter estimates and adequate statistical power. Accounting for an estimated 10% non-response rate due to the challenging circumstances facing NICU families, a total of 350 participants were recruited.

### 2.5 Statistical analysis

All statistical analyses were conducted using SPSS version 25.0 (IBM Corp., Armonk, NY, USA) and AMOS version 24.0. Descriptive statistics were calculated for all demographic and clinical variables, including means, standard deviations, frequencies, and percentages. The distribution of scale scores was examined for normality using skewness and kurtosis statistics; values within ±2.0 were considered acceptable for normality assumptions.

Independent samples t-tests were conducted to compare NICU-PAS scores between first-time and experienced parents. One-way analysis of variance (ANOVA) was used to examine differences in scale scores across demographic categories (e.g., education level, income groups). *post-hoc* comparisons with Bonferroni correction were performed when significant overall effects were identified.

Pearson correlation coefficients were calculated to examine relationships between the NICU-PAS and other study variables for convergent and discriminant validity assessment. Correlation magnitudes were interpreted as small (r = 0.10–0.29), medium (r = 0.30–0.49), or large (r ≥ 0.50) based on conventional guidelines. For all analyses, statistical significance was set at *p* < 0.05 (two-tailed). Effect sizes were reported alongside significance tests to facilitate interpretation of practical significance.

### 2.6 Ethical considerations

The study was conducted in accordance with the Declaration of Helsinki, and approved by the Ethics Committee of Children's Hospital of Soochow University (Approval number 23CS221). Informed consent was obtained from all subjects involved in the study. Participants were assured that their responses would remain confidential, and they were informed of their right to withdraw from the study at any time without penalty.

## 3 Results

### 3.1 Participant characteristics

A total of 350 parents or primary caregivers of infants admitted to the NICU participated in this study between July 15, 2025 and November 7, 2025. Table 1 summarizes the characteristics of the final analytic sample of 350 caregivers. Most respondents were aged 25–39 years, with 89 participants (25.4%) aged 25–29 and 146 participants (41.7%) aged 30–39, while smaller proportions were aged 18–24 (60, 17.1%) and 40 years or older (55, 15.7%). 119 participants (34.0%) were first-time parents, whereas 231 (66.0%) reported prior parenting experience. The sample included slightly more women than men, with 192 female caregivers (54.9%) and 158 male caregivers (45.1%). Educational attainment was generally high, with 191 participants (54.6%) reporting technical college or undergraduate education and 89 (25.4%) reporting postgraduate education or above; 70 participants (20.0%) had senior high school as their highest education.

**Table 1:** Demographic characteristics of the study sample (*N* = 350).

Characteristic	*n*	%
Age (years)		
18–24	60	17.1
25–29	89	25.4
30–39	146	41.7
40+	55	15.7
Gender		
Female	192	54.9
Male	158	45.1
Education Level		
High school	70	20.0
Tech college/Undergraduate	191	54.6
Postgraduate or over	89	25.4
Monthly Household Income (CNY)		
<15,000	132	37.7
15,000–30,000	126	36.0
30,001–50,000	59	16.9
>50,000	33	9.4
Caregiver Relationship		
Mother	178	50.9
Father	140	40.0
Grandparent	24	6.9
Other	8	2.3
Parent Type		
First-time	119	34.0
Experienced	231	66.0

Monthly household income was concentrated in the lower-to-middle ranges, with 132 participants (37.7%) reporting <15,000 CNY and 126 (36.0%) reporting 15,000–30,000 CNY, while 59 (16.9%) reported 30,001–50,000 CNY and 33 (9.4%) reported >50,000 CNY. In terms of relationship to the infant, most respondents were parents, with mothers comprising 178 (50.9%) and fathers 140 (40.0%); grandparents accounted for 24 (6.9%) and other caregivers for 8 (2.3%).

### 3.2 Factor structure

#### 3.2.1 Exploratory factor analysis (EFA)

EFA was conducted to examine the underlying factor structure of the NICU-PAS. The KMO measure of sampling adequacy was 0.844, indicating meritorious suitability for factor analysis. Bartlett's test of sphericity was statistically significant [*χ*^2^(105) = 2081.02, *p* < 0.001], confirming that the correlation matrix was appropriate for factor analysis.

Principal axis factoring with varimax rotation revealed four factors with eigenvalues exceeding 1.0, consistent with the theoretically proposed four-domain structure. Factor 1 (Treatment Understanding) accounted for 31.66% of the variance (eigenvalue = 4.749), Factor 2 (Emotional Well-being) accounted for 13.77% (eigenvalue = 2.065), Factor 3 (Communication) accounted for 12.14% (eigenvalue = 1.821), and Factor 4 (Decision Making) accounted for 10.60% (eigenvalue = 1.591). Collectively, the four factors explained 68.17% of the total variance.

**Figure 1:**
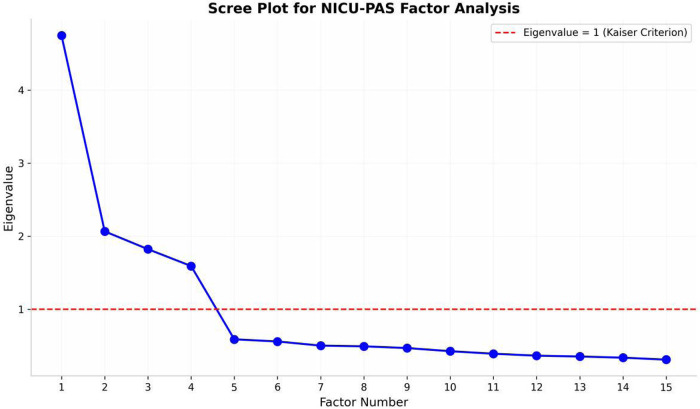
Scree plot for NICU-PAS factor analysis. Scree plot of eigenvalues from the exploratory factor analysis of the 15 NICU-PAS items. The curve shows an inflection after the fourth factor, and only the first four factors exceed the Kaiser criterion (eigenvalue = 1.0), supporting a four-factor structure.

The scree plot (Figure 1) showed a clear inflection after the fourth factor. In addition, only the first four factors had eigenvalues greater than 1.0, supporting retention of a four-factor solution.

**Figure 2:**
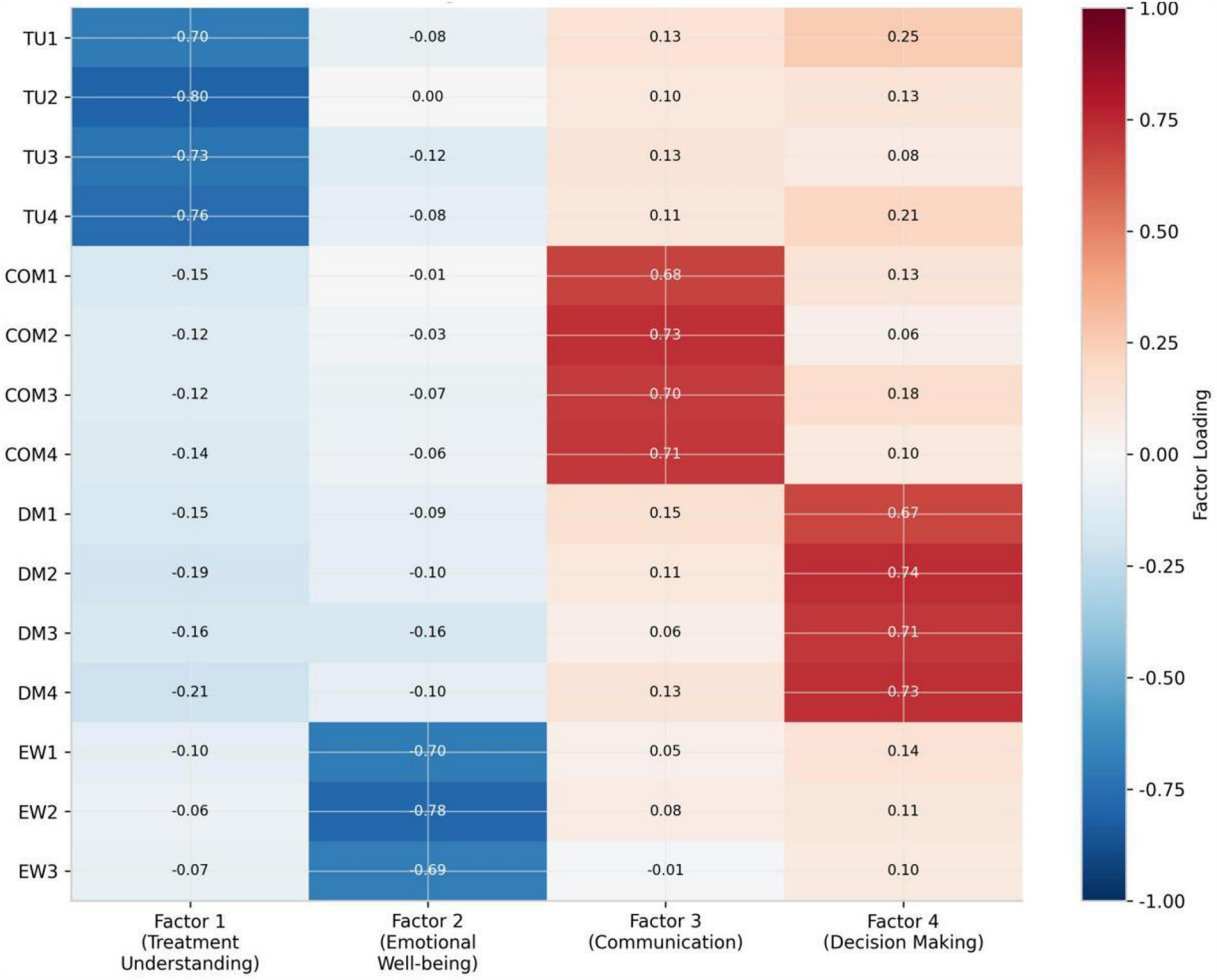
Factor loadings matrix for NICU-PAS items (rotated solution). Heatmap of rotated factor loadings for the NICU-PAS items across four factors. Items show strong primary loadings on their intended domains (Treatment Understanding, Emotional Well-being, Communication, and Decision Making) with minimal cross-loadings, supporting the structural validity of the four-domain model.

Factor loadings are presented in Table 2 and visualized in Figure 2. All items demonstrated strong primary loadings on their intended factors, ranging from 0.67 to 0.80. Cross-loadings were minimal, with no item showing a secondary loading exceeding 0.30 on any unintended factor. This pattern of loadings provides strong evidence for the structural validity of the four-factor model.

**Table 2:** Exploratory factor analysis results.

**Item**	**Factor 1 (TU)**	**Factor 2 (EW)**	**Factor 3 (COM)**	**Factor 4 (DM)**	**Communality**
TU1	**0**.**700**	0.077	0.133	0.252	0.583
TU2	**0**.**804**	0.001	0.104	0.129	0.673
TU3	**0**.**734**	0.122	0.126	0.084	0.577
TU4	**0**.**759**	0.080	0.112	0.209	0.637
EW1	0.098	**0**.**702**	0.049	0.137	0.514
EW2	0.057	**0**.**783**	0.075	0.114	0.633
EW3	0.073	**0**.**690**	0.009	0.099	0.487
COM1	0.151	0.007	**0**.**677**	0.130	0.491
COM2	0.118	0.033	**0**.**729**	0.058	0.548
COM3	0.120	0.068	**0**.**696**	0.178	0.531
COM4	0.136	0.057	**0**.**709**	0.098	0.532
DM1	0.151	0.088	0.150	**0**.**669**	0.504
DM2	0.188	0.096	0.108	**0**.**738**	0.594
DM3	0.160	0.159	0.063	**0**.**711**	0.560
DM4	0.210	0.095	0.133	**0**.**730**	0.596
Eigenvalue	4.749	2.065	1.821	1.591	
% Variance	31.66	13.770	12.140	10.600	

Factor loadings > 0.40 are shown in bold (indicated here by factor assignment). TU, treatment understanding; EW, emotional well-being; COM, Communication; and DM, decision making. KMO = 0.844; Bartlett's test *χ*^2^(105) = 2081.02, *p* < 0.001.

#### 3.2.2 Confirmatory factor analysis

Confirmatory factor analysis was performed to validate the four-factor structure identified through EFA. Model fit indices are presented in Table 3. The chi-square statistic was χ^2^(84) = 142.35, with a chi-square to degrees of freedom ratio of 1.69, indicating acceptable model fit. The comparative fit index (CFI) was 0.972 and the Tucker–Lewis index (TLI) was 0.965, both exceeding the recommended threshold of 0.95 for good model fit.

**Table 3:** Confirmatory factor analysis model Fit indices.

**Index**	**Value**	**Criterion**	**Model Fit**
Chi-square (*χ*^2^)	142.35	NA	NA
Degrees of freedom (df)	84	NA	NA
χ^2^/df	1.69	< 3.0	Good
CFI	0.972	> 0.95	Good
TLI	0.965	> 0.95	Good
RMSEA	0.045	< 0.08	Good
RMSEA 90% CI	[0.032, 0.058]	< 0.08	Good
SRMR	0.041	< 0.08	Good

CFI, comparative fit index; TLI, Tucker–Lewis Index; RMSEA, root mean square error of approximation; SRMR, standardized root mean square residual.

Absolute fit indices further supported the model's adequacy. The root mean square error of approximation (RMSEA) was 0.045, with a 90% confidence interval of 0.032–0.058, well below the recommended cutoff of 0.06. The standardized root mean square residual (SRMR) was 0.041, below the threshold of 0.08. All standardized factor loadings were statistically significant (*p* < 0.001) and ranged from 0.65 to 0.82, indicating strong relationships between items and their respective latent factors.

### 3.3 Descriptive statistics and reliability

Descriptive statistics for the NICU-PAS domains and total scale are presented in Table 4. Mean scores across all four domains were remarkably consistent, ranging from 3.49 to 3.51 on the 5-point Likert scale. Treatment Understanding yielded a mean of 3.51 (SD = 0.57), Communication a mean of 3.51 (SD = 0.60), Decision Making a mean of 3.49 (SD = 0.57), and Emotional Well-being a mean of 3.50 (SD = 0.58). The overall scale mean was 3.50 (SD = 0.28), with scores ranging from 2.75 to 4.15. These findings indicate that parents generally reported moderate to high levels of perceived agency across all domains during their infants' NICU hospitalization.

**Table 4:** Descriptive statistics and internal consistency of NICU-PAS domains.

Domain	Mean	SD	*α*	Interpretation
Treatment Understanding	3.51	0.57	0.860	Good
Communication	3.51	0.60	0.815	Good
Decision Making	3.49	0.57	0.837	Good
Emotional Well-being	3.50	0.58	0.783	Acceptable
Total Scale	3.50	0.28	0.839	Good

All items rated on a 5-point Likert scale (1 = strongly disagree, 5 = strongly agree). α = Cronbach's alpha.

**Figure 3:**
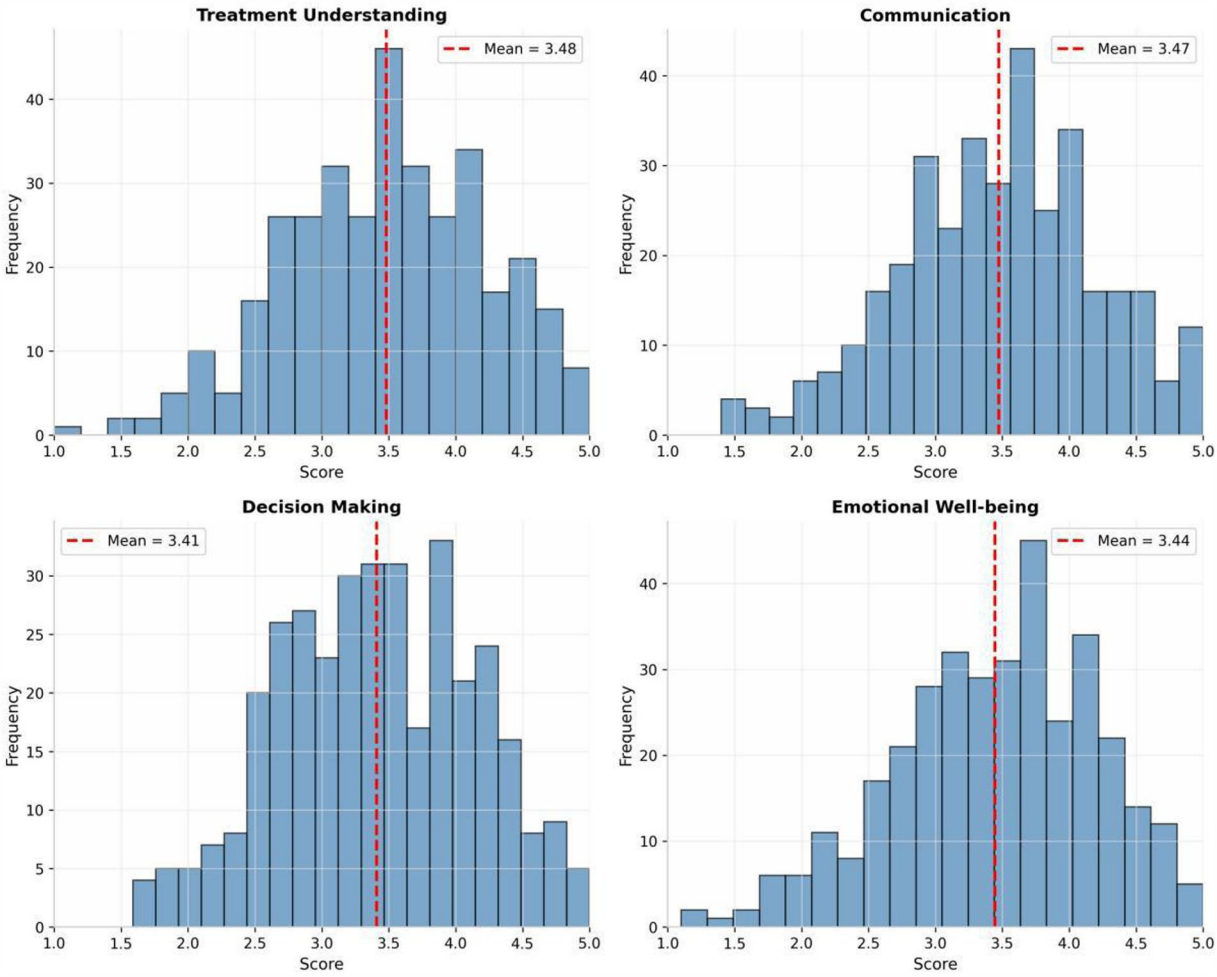
Distribution of NICU-PAS domain scores. Histograms showing the distribution of domain scores for Treatment Understanding, Communication, Decision Making, and Emotional Well-being. Dashed vertical lines indicate mean domain scores, demonstrating approximately normal score distributions across domains.

The distributions of domain scores are illustrated in Figure 3, which demonstrates the relatively normal distribution of responses across all four subscales. Standard deviations were similar across domains (ranging from 0.57 to 0.60), suggesting comparable variability in responses. The narrow range of total scale scores (SD = 0.28) reflects the internal consistency of the aggregated measure.

Internal consistency reliability was assessed using Cronbach's alpha coefficients for each domain and the overall scale. As shown in Table 2, all domains demonstrated acceptable to good internal consistency. Treatment Understanding achieved the highest reliability (*α* = 0.860), followed by Decision Making (α = 0.837), Communication (α = 0.815), and Emotional Well-being (α = 0.783). The overall scale reliability was good (α = 0.839), exceeding the recommended threshold of 0.70 for research purposes.

Item-total correlations were examined to evaluate the contribution of individual items to their respective domains. All item-total correlations exceeded 0.59, indicating that each item contributed meaningfully to its domain. No items showed correlations below the acceptable threshold, supporting the retention of all 15 items in the final scale.

### 3.4 Validity analysis

#### 3.4.1 Convergent validity

Convergent validity was assessed by examining correlations between NICU-PAS scores and the NICU Parental Stress Scale (NICU-PS). As presented in Table 5, a significant negative correlation was observed between NICU-PAS total scores and NICU-PS scores (r = −0.189, *p* < 0.001). This finding indicates that higher levels of parental agency are associated with lower levels of parental stress, supporting the convergent validity of the scale.

**Table 5:** Correlations for convergent and discriminant validity.

Variable	1	2	3	4
1. NICU-PAS Total	1	NA	NA	NA
2. NICU-PS	−0.189**	1	NA	NA
3. General Health	−0.048	0.052	1	NA
4. Personal Coping	−0.030	−0.015	0.089	1

NICU-PS, NICU Parental Stress Scale.

** *p* < 0.001.

#### 3.4.2 Discriminant validity

Discriminant validity was evaluated by examining correlations between NICU-PAS total scores and theoretically unrelated constructs. As shown in Table 5, no significant correlations were found between the NICU-PAS and general health ratings (*r* = −0.048, *p* = 0.373) or personal coping strategies (*r* = −0.030, *p* = 0.574). The absence of significant relationships with these distinct constructs supports the discriminant validity of the NICU-PAS.

### 3.5 Subgroup comparisons

#### 3.5.1 First-time vs. experienced parents

**Figure 4:**
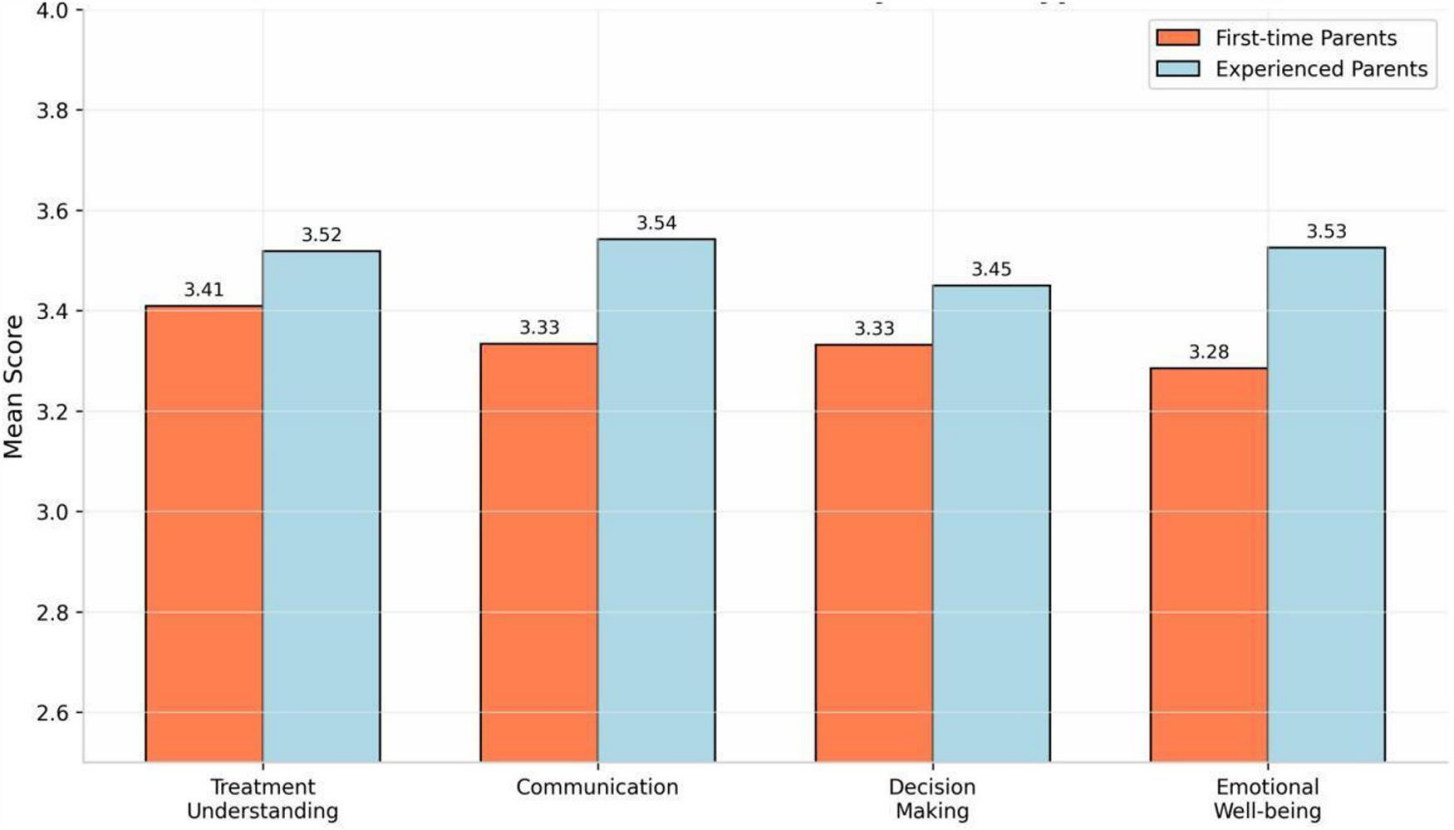
NICU-PAS domain scores by parent type. Comparison of mean NICU-PAS domain scores between first-time parents and experienced parents. Experienced parents report higher mean scores across domains, with significant differences observed for Communication and Emotional Well-being.

Independent samples t-tests were conducted to compare NICU-PAS scores between first-time parents (*n* = 119) and experienced parents (*n* = 231). (Table 6, Figure 4) Experienced parents reported significantly higher scores on Communication [*t*(348) = −2.502, *p* = 0.013], Emotional Well-being [t(348) = −2.852, *p* = 0.005], and total NICU-PAS scores [*t*(348) = −3.054, *p* = 0.002]. No significant differences were observed for Treatment Understanding [*t*(348) = −1.294, *p* = 0.196] or Decision Making [*t*(348) = −1.433, *p* = 0.153].

**Table 6:** Comparison of NICU-PAS scores between first-time and experienced parents.

Domain	First-time (*n* = 119)	Experienced (*n* = 231)	*t*	*p*	*Cohen's d*
Treatment Understanding	3.41 ± 0.73	3.52 ± 0.76	−1.294	0.196	0.146
Communication	3.33 ± 0.74	3.54 ± 0.74	−2.502	0.013*	0.282
Decision Making	3.33 ± 0.78	3.45 ± 0.70	−1.433	0.153	0.162
Emotional Well-being	3.28 ± 0.74	3.53 ± 0.75	−2.852	0.005*	0.322
Total Score	3.34 ± 0.51	3.51 ± 0.48	−3.054	0.002*	0.344

Positive Cohen's d values indicate higher scores for experienced parents. Effect size interpretation: negligible (<0.2), small (0.2–0.5), medium (0.5–0.8), large (≥0.8).

*p < 0.05.

Effect sizes, calculated as Cohen's *d*, ranged from 0.146 to 0.344, indicating negligible to small effect sizes for the significant differences. While statistically significant differences were detected for three outcomes, the practical significance of these differences is limited given the small effect sizes.

#### 3.5.2 Inter-domain correlations

Correlations among the four NICU-PAS domains are presented in Table 7. All inter-domain correlations were positive and statistically significant, ranging from *r* = 0.120 (Communication with Emotional Well-being, *p* < 0.05) to *r* = 0.399 (Treatment Understanding with Decision Making, *p* < 0.01). The moderate magnitude of these correlations suggests that while the domains are related, they represent distinct aspects of parental agency.

**Table 7:** Inter-domain correlations of NICU-PAS.

Domain	1. TU	2. COM	3. DM	4. EW
1. Treatment Understanding (TU)	1.000	NA	NA	NA
2. Communication (COM)	0.303**	1.000	NA	NA
3. Decision Making (DM)	0.399**	0.289**	1.000	NA
4. Emotional Well-being (EW)	0.187**	0.120*	0.264**	1.000

All domains show moderate positive correlations, indicating they measure related but distinct aspects of parental agency.

*p < 0.05, **p < 0.001.

#### 3.5.3 Demographic comparisons

One-way ANOVAs were conducted to examine differences in NICU-PAS total scores across demographic variables. No significant differences were found for education level [F(2, 347) = 0.071, *p* = 0.931], household income [F(3, 346) = 1.954, *p* = 0.121], or caregiver relationship [F(3, 346) = 1.500, *p* = 0.214]. These findings indicate that parental agency, as measured by the NICU-PAS, is relatively consistent across diverse demographic backgrounds, supporting the scale's applicability to a broad range of NICU families.

## 4 Discussion

We developed and psychometrically evaluated the NICU-PAS, a 15-item instrument intended to capture perceived parental agency during an infant’s NICU hospitalization across four domains: Treatment Understanding, Communication, Decision Making, and Emotional Well-being. In a multi-center sample of 350 caregivers recruited from eight participating NICUs in Suzhou, the NICU-PAS demonstrated a coherent four-domain structure supported by both exploratory and confirmatory factor analytic evidence, alongside acceptable to good internal consistency across domains and the total score. Domain means clustered around the midpoint-to-upper range of the 5-point response format and showed approximately normal distributions, suggesting adequate variability for research use. Evidence for construct validity was mixed in an expected way: NICU-PAS scores were inversely related to NICU parental stress but with a small magnitude, showed negligible associations with general health and coping indicators used for discriminant testing, and revealed modest but significant positive inter-domain correlations consistent with related but distinct facets of agency. Subgroup comparisons showed higher scores among experienced parents than first-time parents on Communication, Emotional Well-being, and total score, though effect sizes were small. Taken together, findings support the NICU-PAS as a brief, multidimensional measure of NICU-specific parental agency suitable for research and potentially for quality improvement contexts.

A central contribution of the NICU-PAS is its domain organization around actionable, care-process elements of agency rather than around symptom burden or unit stressors. Many NICU caregiver measures prioritize stress exposure, psychological symptoms, satisfaction, or family-centered care perceptions (15, 21, 26). While valuable, such measures do not always distinguish the caregiver's perceived capability to understand treatment, communicate with clinicians, participate in decisions, and maintain emotional steadiness as separable components (1, 5, 29). The four-factor structure observed here aligns with a conceptualization of agency as a multi-component construct that spans informational, relational, participatory, and affective capacities (1, 5). In the EFA, four factors exceeded eigenvalue criteria, and the scree pattern supported a four-factor solution, a result further reinforced by strong CFA fit indices. Such convergence across EFA and CFA provides a persuasive argument for structural validity in the studied context, particularly because the model fit was strong across both incremental and absolute fit indices.

Internal consistency reliability also compared favorably with the performance typically reported for brief psychosocial and care-process scales in acute care settings (28). Domain alphas fell within acceptable to good ranges, and the total score alpha supported aggregation when a single summary indicator is needed (16). Item-total correlations exceeded conventional minimum thresholds by a wide margin, implying that individual items were strongly aligned with their intended domain content and that scale refinement yielded a cohesive item set. A practical implication is that the NICU-PAS can be deployed without excessive respondent burden, which matters in NICU contexts where families experience time pressure, fatigue, and emotional strain (21, 26). At the same time, the scale retains enough specificity to inform domain-targeted interpretation, which may be more actionable than a single global index (19).

Mean domain scores were tightly clustered, with all domains near 3.5 on a 5-point scale. Such clustering can be interpreted in at least two non-exclusive ways. First, it may reflect a genuine balance in caregivers' perceived agency, where informational, communicative, decisional, and emotional components co-develop during hospitalization (29). Second, it may reflect shared contextual constraints and supports across participating NICUs, for example, standardized communication routines or similar visitation and caregiving arrangements, producing relatively uniform experiences across domains (22, 30). The observed domain distributions were approximately normal, which supports parametric analysis and indicates the measure did not collapse toward extremes in the sampled population. The narrower dispersion for the total score relative to domain scores is expected under aggregation and suggests that domain-level reporting may be particularly informative for detecting differences across people or settings (28).

Evidence for convergent validity emerged through the inverse association between NICU-PAS total score and NICU parental stress, but the correlation magnitude was small. Such a pattern is not a weakness by default. Agency and distress are theoretically related yet non-identical (23). A caregiver can feel worried or exhausted while still perceiving strong capacity to understand treatment plans or communicate effectively with clinicians (21, 23). Conversely, low distress does not guarantee high engagement or shared decision involvement (20). Therefore, a modest negative association can be interpreted as supportive of convergent validity without implying redundancy with stress measures (28). The discriminant validity pattern was also coherent: negligible associations with general health and the coping indicator used for comparison suggest the NICU-PAS is not simply indexing global wellness orientation or generic coping style, but rather a NICU-situated perception of agency tied to care interaction and participation (28).

Inter-domain correlations were significant and modest in size, supporting a model in which domains share common variance while remaining distinct. The strongest association between Treatment Understanding and Decision Making is conceptually consistent with the notion that decisional participation typically presupposes comprehension of clinical information, risks, and tradeoffs (20, 22). The weakest association between Communication and Emotional Well-being, while still significant, suggests that effective interaction with staff may not fully buffer emotional strain, particularly in a high-stakes environment where uncertainty and fear can persist despite adequate communication (21, 23).

Subgroup results indicated that experienced parents reported higher Communication, Emotional Well-being, and total scores than first-time parents, with small effect sizes (31, 32). Prior experience with pediatric hospitalization or comparable healthcare encounters may be more directly relevant than parenting experience alone, because such experience can familiarize caregivers with clinical routines, medical communication, uncertainty, and the need to ask questions during time-sensitive care (31, 33, 34). Notably, differences were not observed for Treatment Understanding or Decision Making, which may be shaped more by how clinicians present information and structure decisions than by parental experience alone (35, 36). In practical terms, the small effect sizes suggest that first-time parent status may function as an imperfect proxy for prior healthcare-related experience and should not be used for risk stratification by itself (36). Instead, first-time status could serve as an initial flag for offering communication coaching, anticipatory guidance, and emotional support, while still assessing individual needs directly (32, 33).

Null findings across education, income, and caregiver relationship for total NICU-PAS scores are also noteworthy. Prior literature often reports socioeconomic gradients in health literacy, communication confidence, and participation (37–39). The absence of detected differences here may indicate that unit-level practices reduced disparities, or it may reflect limited sensitivity given the education distribution and the fact that a total score can mask domain-specific variation. Another possibility is that the scale targets perceived agency within the current care environment, which can be shaped strongly by staff behavior, clarity of information, and inclusiveness of decision routines (35, 36). Under that framing, supportive NICU practices could compress demographic differences even when broader structural inequities remain. Future work should examine domain-level differences and potential moderation by site-level policies to clarify interpretation (36).

### 4.1 Limitations and implications

Several limitations should be considered. Convenience sampling may introduce selection bias, and participation from eight NICUs within a single city supports breadth across local units but does not justify strong inference to other regions or health systems. The design was cross-sectional, so temporal stability and sensitivity to change were not assessed, limiting conclusions about responsiveness for intervention evaluation. Validity evidence relied on correlations with NICU parental stress and brief external indicators, and future work should add stronger criterion measures such as anxiety, depression, trauma symptoms, health literacy, and observed engagement behaviors. EFA and CFA were conducted within the same overall sample, which can inflate apparent confirmation; an external validation sample would strengthen evidence for structural stability. Practical implications remain meaningful. The NICU-PAS offers a brief way to quantify caregiver agency across care-process domains that map onto modifiable clinical practices. Domain-level use could guide targeted supports, for example, structured information tools to strengthen Treatment Understanding, communication coaching to enhance Communication, shared decision prompts to support Decision Making, and screening with referral pathways aligned to Emotional Well-being.

### 4.2 Future directions and clinical relevance

Next steps should include replication across geographically diverse NICUs and across differing visitation and caregiving policies, since agency may be shaped by access, communication cadence, and opportunities for participation. Test-retest reliability and longitudinal trajectories should be evaluated, including whether scores shift with length of stay, clinical acuity, or major treatment transitions. Measurement invariance testing across parent type, caregiver relationship, and demographic groups would clarify whether comparisons reflect true differences or measurement artifacts. Intervention studies could evaluate whether structured family-centered communication programs, shared decision aids, or psychoeducational packages measurably increase domain scores and whether such increases link to downstream outcomes such as reduced decisional conflict, better comprehension, improved satisfaction with communication, or lower stress over time. At the clinical level, NICU teams may benefit from treating parental agency as a care quality target, not merely as an individual trait. A measure that separates informational, relational, participatory, and emotional facets can help teams identify where support is needed and where system design, rather than caregiver characteristics, is the primary lever for improvement.

## 5 Conclusions

The NICU-PAS demonstrates strong pragmatic and psychometric properties. The instrument is concise, theoretically grounded, and organized into four clinically salient domains, supporting feasibility for use in NICU settings. The observed internal consistency and the empirically supported four-factor structure, together with excellent confirmatory model fit, provide strong evidence for reliability and structural validity. Construct validity is further supported by the expected inverse association with NICU-related parental stress and negligible associations with less theoretically proximal constructs. The instrument provides a sound measure of parental agency during NICU hospitalization and may be suitable for research applications, quality improvement, and domain-specific assessment in clinical practice.

## Data Availability

The raw data supporting the conclusions of this article will be made available by the authors, without undue reservation.
