# Supplementary material for: Development and psychometric validation of the NICU parental agency scale: a multicenter cross-sectional study
Source: Front Pediatr. 2026 Jul 27;14:1842011. doi: 10.3389/fped.2026.1842011 (PMC13454491; doi:10.3389/fped.2026.1842011)
Supplement: Supplementary file 1 [file Datasheet1.pdf]

Appendix A: NICU Parental Agency Scale (NICU-PAS v6.4)

Instructions: These items relate to your experience in the NICU. Please respond based on your experiences over the past seven days; if the hospitalization has been shorter than seven days, respond based on your experience since admission. Please answer honestly.

Scoring: Each item is scored on a 5-point Likert scale: 1 = Strongly Disagree, 2 = Disagree, 3 = Neutral/Unsure, 4 = Agree, 5 = Strongly Agree. Higher scores indicate higher perceived parental agency.

Domains and Items:

1. Treatment Understanding NICUPAS_01: I am able to understand the healthcare providers' treatment plan for my infant. NICUPAS_02: I am able to understand changes in my infant's condition. NICUPAS_03: I clearly understand the treatment measures and plan for my infant.

2. Communication and Feedback NICUPAS_04: I understand the content communicated by healthcare providers and can respond appropriately. NICUPAS_05: I can ask questions when healthcare providers provide updates. NICUPAS_06: If I do not understand something, I can ask healthcare providers for further explanation. NICUPAS_07: I can clearly express my concerns about my infant's condition. NICUPAS_08: I feel that healthcare providers respect my opinions and feelings during my infant's treatment.

3. Care Participation and Collaboration NICUPAS_09: I believe I can play an active role in my infant's care. NICUPAS_10: I clearly understand how my participation contributes to my infant's care. NICUPAS_11: I feel I am an important partner in my infant's care process. NICUPAS_12: Healthcare providers respond fully when I ask questions. NICUPAS_13: Healthcare providers are patient and willing to explain during communication.

4. Decision Participation and Understanding NICUPAS_14: Healthcare providers listen carefully and provide feedback when I raise opinions or concerns. NICUPAS_15: I feel that I can fully understand the advantages and disadvantages of treatment plans and participate in decisions.

Domain Scores: Treatment Understanding = mean(NICUPAS_01, NICUPAS_02, NICUPAS_03) Communication and Feedback = mean(NICUPAS_04, NICUPAS_05, NICUPAS_06, NICUPAS_07, NICUPAS_08) Care Participation and Collaboration = mean(NICUPAS_09, NICUPAS_10, NICUPAS_11, NICUPAS_12, NICUPAS_13) Decision Participation and Understanding = mean(NICUPAS_14, NICUPAS_15)

Total NICU-PAS Score = mean(NICUPAS_01 to NICUPAS_15)

Optional 0–100 transformation: (Mean Score - 1) / 4 * 100

Minimum data requirements for scoring:

- Total score: ≥13 of 15 items completed

- Treatment Understanding: ≥2 of 3 items completed
- Communication and Feedback: ≥4 of 5 items completed
- Care Participation and Collaboration: ≥4 of 5 items completed
- Decision Participation and Understanding: both items completed

NICU 患儿家属能动性量表（NICU-PAS v6.4）

说明：以下题目与您在 NICU 的体验有关，请根据过去 7 天内的经历作答；如住院不足 7 天，请按入 NICU 以来的经历作答。请根据您的真实感受作答。

计分 每题使用 5 点 Likert 评分：1=非常不同意，2=不同意，3=一般/说不清，4=同意，5=非常同意。得分越高，表示父母在 NICU 中感知的能动性越高。

量表维度与条目

一、治疗计划与病情理解 NICUPAS_01：我能理解医护人员对宝宝的治疗计划。NICUPAS_02：我能够理解宝宝的病情变化。 NICUPAS_03：我能清楚地理解宝宝的治疗措施和治疗计划。

二、沟通与反馈 NICUPAS_04：我能理解医护人员的沟通内容并做出相应反应。NICUPAS_05：我能够在医护人员提供病情更新时提出问题。 NICUPAS_06：如果我不理解某些内容，我能够请求医护人员进行进一步解释。 NICUPAS_07：我能清晰地表达自己对宝宝病情的担忧。 NICUPAS_08：我感到在宝宝的治疗过程中，医护人员尊重我的意见和感受。

三、照护参与与合作 NICUPAS_09：我认为自己可以在宝宝护理中起到积极的作用。 NICUPAS_10：我能清楚地知道我的参与对宝宝的照护有何帮助。NICUPAS_11：我感到自己是宝宝护理过程中的重要合作伙伴。 NICUPAS_12：当我提出问题时，医护人员会给予充分的回应。 NICUPAS_13：在与医护人员沟通时，我感到他们耐心并愿意解释。

四、决策参与与理解 NICUPAS_14：当我提出意见或担忧时，医护人员会认真听取并给予反馈。 NICUPAS_15：我觉得自己能够充分理解治疗方案的利弊，并参与决策。

分量表计分 治疗计划与病情理解 = 均值(NICUPAS_01, NICUPAS_02, NICUPAS_03) 沟通与反馈 = 均值(NICUPAS_04, NICUPAS_05, NICUPAS_06, NICUPAS_07, NICUPAS_08) 照护参与与合作 = 均值(NICUPAS_09, NICUPAS_10, NICUPAS_11, NICUPAS_12, NICUPAS_13) 决策参与与理解 = 均值(NICUPAS_14, NICUPAS_15)

总分 NICU-PAS = 均值(NICUPAS_01 至 NICUPAS_15)

可选 0–100 转换：(均值 - 1) / 4 * 100

计分最小完成要求：

- 总分：至少 13 项完成
- 治疗计划与病情理解：至少 2/3 项完成
- 沟通与反馈：至少 4/5 项完成
- 照护参与与合作：至少 4/5 项完成
- 决策参与与理解：2 项均完成
